# Multi-omics strategies uncover the molecular mechanisms of nitrogen, phosphorus and potassium deficiency responses in *Brassica napus*

**DOI:** 10.1186/s11658-023-00479-0

**Published:** 2023-08-05

**Authors:** Ying Fu, Annaliese S. Mason, Maolin Song, Xiyuan Ni, Lei Liu, Jianghua Shi, Tanliu Wang, Meili Xiao, Yaofeng Zhang, Donghui Fu, Huasheng Yu

**Affiliations:** 1 Institute of Crop and Nuclear Technology Utilization, Zhejiang Academy of Agricultural Sciences, Hangzhou, China; 2 College of Advanced Agricultural Sciences, Zhejiang A&F University, Hangzhou, China; 3 Plant Breeding Department, University of Bonn, Katzenburgweg 5, 53115 Bonn, Germany; 4 Key Laboratory of Crop Physiology, Ecology and Genetic Breeding, Ministry of Education, Agronomy College, Jiangxi Agricultural University, Nanchang, 330045 China

**Keywords:** Nitrogen, Phosphorus, Potassium, Deprivation, RNA, Metabolite

## Abstract

**Background:**

Nitrogen (N), phosphorus (P) and potassium (K) are critical macronutrients in crops, such that deficiency in any of N, P or K has substantial effects on crop growth. However, the specific commonalities of plant responses to different macronutrient deficiencies remain largely unknown.

**Methods:**

Here, we assessed the phenotypic and physiological performances along with whole transcriptome and metabolomic profiles of rapeseed seedlings exposed to N, P and K deficiency stresses.

**Results:**

Quantities of reactive oxygen species were significantly increased by all macronutrient deficiencies. N and K deficiencies resulted in more severe root development responses than P deficiency, as well as greater chlorophyll content reduction in leaves (associated with disrupted chloroplast structure). Transcriptome and metabolome analyses validated the macronutrient-specific responses, with more pronounced effects of N and P deficiencies on mRNAs, microRNAs (miRNAs), circular RNAs (circRNAs) and metabolites relative to K deficiency. Tissue-specific responses also occurred, with greater effects of macronutrient deficiencies on roots compared with shoots. We further uncovered a set of common responders with simultaneous roles in all three macronutrient deficiencies, including 112 mRNAs and 10 miRNAs involved in hormonal signaling, ion transport and oxidative stress in the root, and 33 mRNAs and 6 miRNAs with roles in abiotic stress response and photosynthesis in the shoot. 27 and seven common miRNA-mRNA pairs with role in miRNA-mediated regulation of oxidoreduction processes and ion transmembrane transport were identified in all three macronutrient deficiencies. No circRNA was responsive to three macronutrient deficiency stresses, but two common circRNAs were identified for two macronutrient deficiencies. Combined analysis of circRNAs, miRNAs and mRNAs suggested that two circRNAs act as decoys for miR156 and participate in oxidoreduction processes and transmembrane transport in both N- and P-deprived roots. Simultaneously, dramatic alterations of metabolites also occurred. Associations of RNAs with metabolites were observed, and suggested potential positive regulatory roles for tricarboxylic acids, azoles, carbohydrates, sterols and auxins, and negative regulatory roles for aromatic and aspartate amino acids, glucosamine-containing compounds, cinnamic acid, and nicotianamine in plant adaptation to macronutrient deficiency.

**Conclusions:**

Our findings revealed strategies to rescue rapeseed from macronutrient deficiency stress, including reducing the expression of non-essential genes and activating or enhancing the expression of anti-stress genes, aided by plant hormones, ion transporters and stress responders. The common responders to different macronutrient deficiencies identified could be targeted to enhance nutrient use efficiency in rapeseed.

**Supplementary Information:**

The online version contains supplementary material available at 10.1186/s11658-023-00479-0.

## Introduction

Nitrogen (N), phosphorus (P) and potassium (K) represent critical macronutrients for growth and development in plants. These nutrients play specific roles in fundamental metabolic pathways, and are associated with diverse morphophysiological aspects of plant growth and development [1]. N is an important element in cellular molecules, including plant proteins, nucleic acids, adenosine triphosphate (ATP), chlorophyll, alkaloids and plant hormones [2]. P is a key structural element that participates in many biological and metabolic processes, including membrane and nucleotide syntheses, energy transmission, signal transduction and photosynthesis [3]. K is involved in enzyme activation, osmotic balance, pH regulation in cells, photosynthesis, stomatal movements and cellular catioN–anion balance, and also influences the mechanical stability of primary and secondary metabolites [4]. In crops, deprivation of macronutrients critically limits plant growth and development. Therefore, unveiling the mechanisms by which crops sense and adapt to nutrient deprivation is essential in improving nutrient utilization, crop yield and quality traits [5, 6].

Many physiological and biochemical changes are induced in response to macronutrient deficiency stresses in plants. The roots of higher plants represent the first and primary sites of nutrient perception and uptake. In plants, the root system architecture is modulated based on varying soil nutrient concentrations [7]. In addition, plants employ various signals, including reactive oxygen species (ROS), sugars, phytohormones, calcium, phosphatidic acid, and nutrient responsive transcription factors/genes for maintaining nutrient homeostasis under macronutrient starvation conditions [8, 9]. These signals initiate substantial alterations in the metabolic, physiological and hormonal pathways in response to macronutrient deficiency, and affected processes such as nutrient utilization, chlorophyll biosynthesis/photosynthesis, anthocyanin biosynthesis and hormone biosynthesis [10, 11].

A number of key genes have been reported for plant response to N, P and K deficiency stresses. With respect to nitrogen sensing and transport, four protein groups, i.e., nitrate transporter 1 (NRT1)/peptide transporter (PTR) family (NPF), nitrate transporter 2 (NRT2), chloride channel (CLC), and slowly activating anion channel (SLAC) families, have been implicated [12]. The transcription factor *AtNLP7* is considered a critical inducer of primary nitrate response in Arabidopsis [13]. Further genes with diverse functions, for example sensors, transcription factors, protein kinases and polyadenylation specificity factors, compose the molecular networks that coordinate responses to N availability in Arabidopsis and rice [14, 15]. Phosphorous deprivation conditions have been shown to activate genes such as *P starvation tolerance 1* (*PSTOL1*), *actin-related protein 6* (*ARP6*), *plastid transcription factor 1* (*PTF1*), *purple acid phosphatases* (*PAPs*), *phosphate 1* (*PHO1*), *induced by phosphate starvation 1* (*IPS1*), *phosphate transporters* (*PHTs*), *ribonucleases* (*RNSs*), *UDP sulfoquinovose synthases* (*SQDs*), and *phospholipase DZ2* (*PLDZ2*) [16, 17]. Potassium deprivation initiates responses in a number of K channels (e.g. *Shaker*, *TPK*, and *Kir-like*) and transporters (e.g. *KUP/HAK/KT, HKT* and *CPA*) [18–22].

Non-coding RNAs and metabolites are also involved in plant response to nutrient deficiencies. Many microRNAs (miRNAs) responsive to nutrient stress have been identified, e.g. N-responsive miR166, miR167, miRNA169, miR393, miR444, miR826, miR827 and miR5090; P-responsive miR156, miR159, miR166, miR169, miR319, miR395, miR398, miR399, miR447, and miR827 [23]; and K-responsive miR319, miR396, miR408 and miR444 [24]. Of these, only miR444a responds to each of N, P, and K stresses. Sporadic reports have demonstrated the potential for circular RNA (circRNA) to play a role in response to macronutrient deficiency [25], although this is as yet under-investigated. Bridging phenotypes and genotypes, several metabolites also change significantly in plants after exposure to macronutrient stressors. Under P-deficiency stress, P-rich metabolites are substantially reduced [26]. Under N-deficiency stress, amino acids, such as lysine, tyrosine, threonine, ornithine and glutamine, are also dramatically reduced [27]. K-deficiency stress results in significant increases in sugars and amino acids, especially those that are nitrogen-rich [28]. Although many studies have investigated the individual impact of N, P or K supply on mRNAs, miRNAs or metabolites, a combined multi-omics approach to investigate commonalities in these complex plant responses to macronutrient stresses may provide tools for the improvement of nutrient use efficiency in crops.

Rapeseed (*Brassica napus* L.) represents an important oil crop in temperate zones world-wide. Macronutrient deficiency is a key limiting factor in rapeseed production, as high amounts of NPK fertilizer need to be applied in most environments to optimize productivity [29]. Although several studies have identified responses occurring in plants stressed by N, P or K deprivation individually in rapeseed [26–29], comparative analysis of the combined response/s to different macronutrient deficiencies has seldom been reported. Comparisons between studies assessing different nutrient deficiency responses are also hard to perform because of divergent experimental conditions: studies may vary in plant growth conditions, treatment methodologies, time intervals for sample collection, genotypes used and in microarray/genotyping methods. A more detailed understanding of plant response to N, P, and K deficiencies, and specifically the similarities and differences observed between macronutrient deficiency responses, may provide novel insights into key regulatory pathways and hence breeding targets for increased nutrient use efficiency and tolerance to nutrient deprivation. In the present study, rapeseed seedlings cultured with different N, P or K concentrations were investigated for morphological and physiological differences and characterized at the transcriptomic and metabolomic levels to compare changes in mRNAs, miRNAs, circRNAs and metabolites. Numerous morphological, physiological, ultrastructural, molecular and metabolic changes were detected, providing substantial mechanistic insights and highlighting common and specific features in the molecular, cellular and metabolic responses of rapeseed seedlings to macronutrient deficiencies.

## Materials and methods

### Plant materials and growth conditions

Zhongshuang 11, a semi-winter cultivar with genome sequence information, elite oil quality traits, high seed production and strong stress adaptation was selected as the rapeseed line to be studied for the following experiments. Hydroponic culture was carried out using Hoagland nutrient solution. Seeds underwent surface-sterilization for 5 min with 10% (w/v) sodium hypochlorite followed by three washes with deionized water. This was followed by sowing on moistened gauze that was fixed to a tray filled with deionized water. The sown seeds were grown for five days at 22–24 °C in an illuminated culture room until germination occurred. Uniform seedlings were selected and grown in different nutrient solutions.

Four independent hydroponic culture experiments were designed, including a control (CK), low N (N-), low P (P-), and low K (K-) treatment. Each treatment was carried out in three biological replicates. The full-strength nutrient solution used as a control was made up according to Wang et al., [29], with 3.0 mm NH_4_NO_3_, 0.28 mm Na_2_HPO_4_·12H_2_O, 0.64 mm NaH_2_PO_4_·H_2_O, 2.0 mm KCl, 0.25 mm CaCl_2_·2H_2_O, 2.0 mm MgSO_4_·7H_2_O, 46.0 μm H_3_BO_3_, 9.0 μm MnCl_2_·4H_2_O, 0.3 μm CuSO_4_·5H_2_O, 0.8 μm ZnSO_4_·7H_2_O, 0.1 μm (NH_4_)6Mo_7_O_24_ and 50.0 μm Fe-EDTA. The treatment conditions of N-, P- and K- had concentrations of N, P, and K reduced to one-tenth of that of the full-strength nutrient solution (CK), respectively. The details were as follows: low N had 0.3 mm NH_4_NO_3_, low P had 28 μm Na_2_HPO_4_·12H_2_O and 64 μm NaH_2_PO_4_·H_2_O, and low K had 0.2 mm KCl. Considering the lower nutrition demand of rapeseed seedlings during the early post-germination period, the germinated seedlings were firstly grown in 1/4-strength solutions for five days, followed by 1/2-strength solutions for five days (1/4 and 1/2 strength relative to each of the CK, low N, low P and low K treatments), and then in the final nutrient deprivation solutions (CK, low N, low P, low K). The pH of the nutrient solution was controlled at 5.8–6.0. All plants were grown in an illuminated growth chamber (light intensity: 150 μmol m^− 2^ s^− 1^, temperature: 24 °C day/22 °C night, light period: 16 h photoperiod/8 h dark, relative humidity: 60%) [29].

### Measurements of plant biomass, tissue macronutrient concentrations, and root morphological features

Rapeseed seedlings were harvested at 4 days, 11 days, 18 days and 25 days after hydroponic culture, with three biological replicates for each treatment. The samples underwent washes with distilled water before biomass measurements. Fresh root and shoot samples were weighed. The root morphological features of root length and root diameter were quantified using a WINRHIZO Epson Perfection V700 Photo (JZZIA, Seiko Epson Corp., Japan). Plant root and shoot samples were collected after 28 days in the hydroponic solution to measure the tissue macronutrient concentrations. Samples were washed with deionized water before were measured. The dried specimens were pulverized and submitted to digestion using H_2_SO_4_-H_2_O_2_ as below. Samples were heated with 105 ℃ for 15 min at 75 ℃ until the sample was completely dried, then ground to a particle size of < 0.25 mm. 100 mg samples were weighed out and mixed with 5 mL of concentrated H_2_SO_4_, followed by gentle shaking in a micro Kjeldahl digestion unit for more than eight hours. The resulting suspensions were digested for 30 min at 250 ℃. Subsequently, the temperature was increased to 400 ℃ for three hours. After the suspensions cooled down, ten drops of H_2_O_2_ were added and suspensions reheated and brought to boiling for 5 min. This procedure was repeated three to five times until the suspension was clear. The residual H_2_O_2_ was eliminated by heating for 5–10 min. The resulting solutions were made up to 50 mL and were used for the following measurements of plant total nitrogen, phosphorus and potassium. Plant total nitrogen was determined by an Automatic Kjeldahl Apparatus instrument. Plant phosphorus was measured according to the vanadomolybdate method as follows: 0.5 mL of the above resulting sample solution was combined with dinitrophenol indicator and molybdenum antimony anticolorant to generate phosphorus molybdenum blue, which was assayed by a UV spectrophotometer [26]. Plant potassium was assessed with the Atomic Absorption Spectrophotometer instrument using the digested solution described above.

### Quantification of reactive oxygen species (ROS) and chlorophyll content

Samples grown for 28 days in the hydroponic solution were used for ROS measurements, with at least three biological replicates for each treatment. Roots and shoots were separated and pulverized in liquid N_2_. 0.1 g of the sample was weighed, and 1 mL of 0.01 mol/L phosphate buffered saline (PH 7.2–7.4) extract solution was added for ice bath homogenization. The solution was centrifuged at 4 °C, 8000*g* for 10 min, then the supernatant was taken and placed on ice for testing. The concentrations of four ROS indices (hydrogen peroxide [H_2_O_2_], malondialdehyde [MDA], superoxide dismutase [SOD] and catalase [CAT]) were assayed following established kit protocols for an enzyme-linked immunosorbent assay (Jiangsu Jingmei Biological Technology Co. LTD, China) (kit ID: JM-01088P2 for H_2_O_2;_ JM-110103P2 for SOD; JM-09865P2 for MDA; JM-01084P2 for CAT). The leaf relative chlorophyll content was assayed at 28 days after macronutrient withdrawal using the SPAD-502 m (Konica Minolta, Japan), to produce SPAD values (Soil and Plant Analyzer Development) that are linearly correlated with the amount of chlorophyll present in the leaf and which are hence widely used as a proxy for relative chlorophyll content.

### Transcriptome analysis

RNA sequencing of mRNAs, miRNAs and circular RNAs was performed using plants grown for 28 days in hydroponic solutions. Three biological replicates (comprising individual plants) were taken for each treatment. The collected above-ground and below-ground tissues were separately frozen in liquid nitrogen, followed by storage at − 80 °C until analysis. Total RNA was isolated from 100 mg of frozen tissue using a TriPure kit (Roche, Basel, Switzerland), as directed by the manufacturer. RNA purity and concentration were assayed using a NanoPhotometer^®^ spectrophotometer. RNA integrity and quantity were measured using an RNA Nano 6000 Assay Kit of the Bioanalyzer 2100 system. Libraries were separately constructed as follows: for RNA-seq, ribosomal RNAs were depleted using the RiboMinus™ Plant Kit (Invitrogen, Germany), following which 500 ng of total RNA was used for the libraries constructed using the Truseq Stranded mRNA library preparation kit (Illumina Inc., CA, USA) following the manufacturers’ protocol. miRNA and circRNA sequencing libraries were generated using an NEB Next^®^ Multiplex Small RNA Library Prep Set for Illumina^®^ (NEB E7300L) and an NEBNext Ultra Directional RNA Library Prep Kit for Illumina (NEB E7420) according to the manufacturers’ protocols, respectively. The quality of the libraries was assessed using an Agilent 2100 Bioanalyzer (for mRNAs) and Agilent 5400 system (for miRNAs and circRNAs). All libraries were quantified by qPCR. The libraries were then sequenced on Illumina platforms with PE150 (mRNAs and circRNAs) and SE50 read kits (miRNAs) (Novogene Bioinformatics Technology Co., Ltd., Beijing, China).

For mRNAs, quality control and low-quality read filters were firstly applied. The resulting clean reads were aligned to the *B. napus* Darmor-bzh v4.1 reference genome [30] (http://www.genoscope.cns.fr/brassicanapus/). For small RNAs, filtered clean reads > 18 nt or < 30 nt underwent alignment to unique sequences in Rfam 14.1 (http://www.sanger.ac.uk/software/Rfam) and GenBank non-coding RNA (http://www.ncbi.nlm.nih.gov/) databases. MiRNAs were predicted by alignment with previously reported miRNAs in miRBase 21.0 (http://www.mirbase.org/). psRNATarget (http://plantgrn.noble.org/psRNATarget/) was utilized to predict the relationships between miRNAs and target genes. To identify circular RNAs, the filtered clean reads were mapped to the *B. napus* reference genome Darmor-*bzh* v4.1 (http://www.genoscope.cns.fr/brassicanapus/) with Bowtie2 (v2.2.8; http://bowtiebio.sourceforge.net/bowtie2/index.shtml), and only uniquely mapped reads with two or less mismatches were subsequently analyzed. find_circ (v1.2, https://github.com/marvin-jens/find_circ) and CIRI2 (https://sourceforge.net/projects/ciri/) were utilized to determine candidate circRNAs [31].

The raw data of mRNAs, miRNAs and circRNAs have been deposited in the Genome Sequence Archive (Genomics, Proteomics and Bioinformatics 2021) in National Genomics Data Center (Nucleic Acids Res 2022), China National Center for Bioinformation/Beijing Institute of Genomics, Chinese Academy of Sciences (GSA: CRA011284 for mRNAs; CRA011287 for miRNAs; CRA011286 for circRNAs) that are publicly accessible at https://ngdc.cncb.ac.cn/gsa. Next, we determined fragments per kilobase of transcript per million mapped reads (FPKM), and differentially expressed transcripts were selected by fold change ≥ 2 and a false discovery rate (FDR) < 0.05. Gene Ontology (GO) (http://www.geneontology.org/) was utilized to determine GO terms with significant enrichment. The Kyoto encyclopedia of genes and genomes (KEGG) database (http://www.genome.ad.jp/kegg/) was utilized for pathway enrichment analysis.

### Gene expression validation

Total RNA isolation and quality etermination were performed using the same approach as described above. The first-strand cDNA was reverse-transcribed from total RNA using HiScript^®^ Q RT SuperMix for qPCR (+ gDNA wiper) (Vazyme) according to the manufacturer’s instructions for the validation of mRNA and circRNA. For miRNA, reverse transcription was performed using PrimeScript RT reagent Kit (Perfect Real Time) (Takara) according to the manufacturer’s instructions. qRT-PCR was performed to validate lncRNA, miRNA and mRNA expression profiles in a CFX96 Real-time System (Bio-RAD, USA), with specific primers designed using Primer Premier 5.0 (PREMIER Biosoft Int., Palo Alto, CA, USA) and qPrimerDB (https://biodb.swu.edu.cn/qprimerdb/). ChamQ SYBR qPCR Master Mix (Vazyme) was used for PCR reaction. We selected the housekeeping genes *ACTIN7* and *U6* as controls, as *U6* is the commonly used reference gene of miRNA, and *ACTIN7* is not only widely used as a reference gene across many species including rapeseed as a result of its high, stable expression levels, but also as this gene was previously reported to show stable expression in rapeseed under different nutrient deficiency stresses [32]. Thus, the expression of *ACTIN7* and *U6* were utilized as reference controls to determine the expression of target genes using the 2^−*ΔΔ*Ct^ method. Three biological replicates were performed, and each was repeated with three technical replicates.

### Metabolite extraction and LC–MS/MS analysis

Six biological replicates of each of root and shoot tissues from individual plants were taken for metabolite extraction. 100 mg of each tissue was individually ground with liquid nitrogen and the homogenates were resuspended with pre-chilled 80% methanol by well vortexing. The samples were incubated on ice for 5 min and then were centrifuged at 15,000*g* at 4 °C for 20 min. The supernatant was diluted to a final concentration of 53% methanol using LC–MS grade water. The samples were subsequently transferred to a fresh Eppendorf tube and were then centrifuged at 15,000*g* at 4 °C for 20 min. Finally, the supernatant was injected into the LC–MS/MS system analysis.

UHPLC-MS/MS analyses were performed using a Vanquish UHPLC system (ThermoFisher, Germany) coupled with an Orbitrap Q ExactiveTMHF mass spectrometer (Thermo Fisher, Germany) in Novogene Co., Ltd. (Beijing, China). Samples were injected onto a HypesilGoldcolumn (100 × 2.1 mm, 1.9 μm) using a 17-min linear gradient at a flow rate of 0.2 mL/minute. The eluents for the positive polarity mode were eluent A (0.1% FA in water) and eluent B (methanol). The eluents for the negative polarity mode were eluent A (5 mM ammonium acetate, pH 9.0) and eluent B (methanol).The solvent gradient was set as follows: 2% B, 1.5 min; 2–85% B, 3 min; 85–100% B, 10 min; 100–2% B, 10.1 min; 2% B, 12 min. A Q ExactiveTM HF mass spectrometer was operated in positive/negative polarity mode with a spray voltage of 3.5 kV, capillary temperature of 320 °C, sheath gas flow rate of 35 psi and auxiliary gas flow rate of 10 L/minute, S-lens RF level of 60, and auxiliary gas heater temperature of 350 °C.

Compound Discoverer 3.1 (CD3.1, ThermoFisher) was utilized for raw data processing for further metabolite quantification. The main parameters were set as follows: retention time tolerance, 0.2 min; actual mass tolerance, 5 ppm; signal intensity tolerance, 30%; signal/noise ratio, 3; and minimum intensity. Peak intensities were normalized to the total spectral intensity. The normalized data was used to predict the molecular formula based on additive ions, molecular ion peaks and fragment ions. Peaks were subsequently matched with the mzCloud (https://www.mzcloud.org/), mzVaultand MassListdatabase to obtain accurate qualitative and relative quantitative results. The raw metabolome data have been deposited in the OMIX database in the China National Center for Bioinformation/Beijing Institute of Genomics, Chinese Academy of Sciences (OMIX: OMIX004297) that are publicly accessible at https://ngdc.cncb.ac.cn.

Metabolite annotation used the KEGG, HMDB (https://hmdb.ca/metabolites) and LIPIDMaps (http://www.lipidmaps.org/) databases. Differentially expressed metabolites were determined using the criteria of variable importance in the projection (VIP) > 1 (VIP values were generated in the partial least-squares-discriminant analysis model and represent the contribution to the discrimination of each metabolite ion between groups), fold change > 1.5 or fold change < 0.667 and *P* value < 0.05. The KEGG database was employed for functional analysis based on the retained metabolites. In the metabolic pathway enrichment analysis of differentially expressed metabolites, ratios satisfying x/n > y/N indicated an enriched metabolic pathway, whereas *p* < 0.05 indicated statistically significant enrichment of the metabolic pathway.

## Results

### The morphological and physiological effects of different macronutrient deficiencies are distinct or opposite

**Fig. 1:**
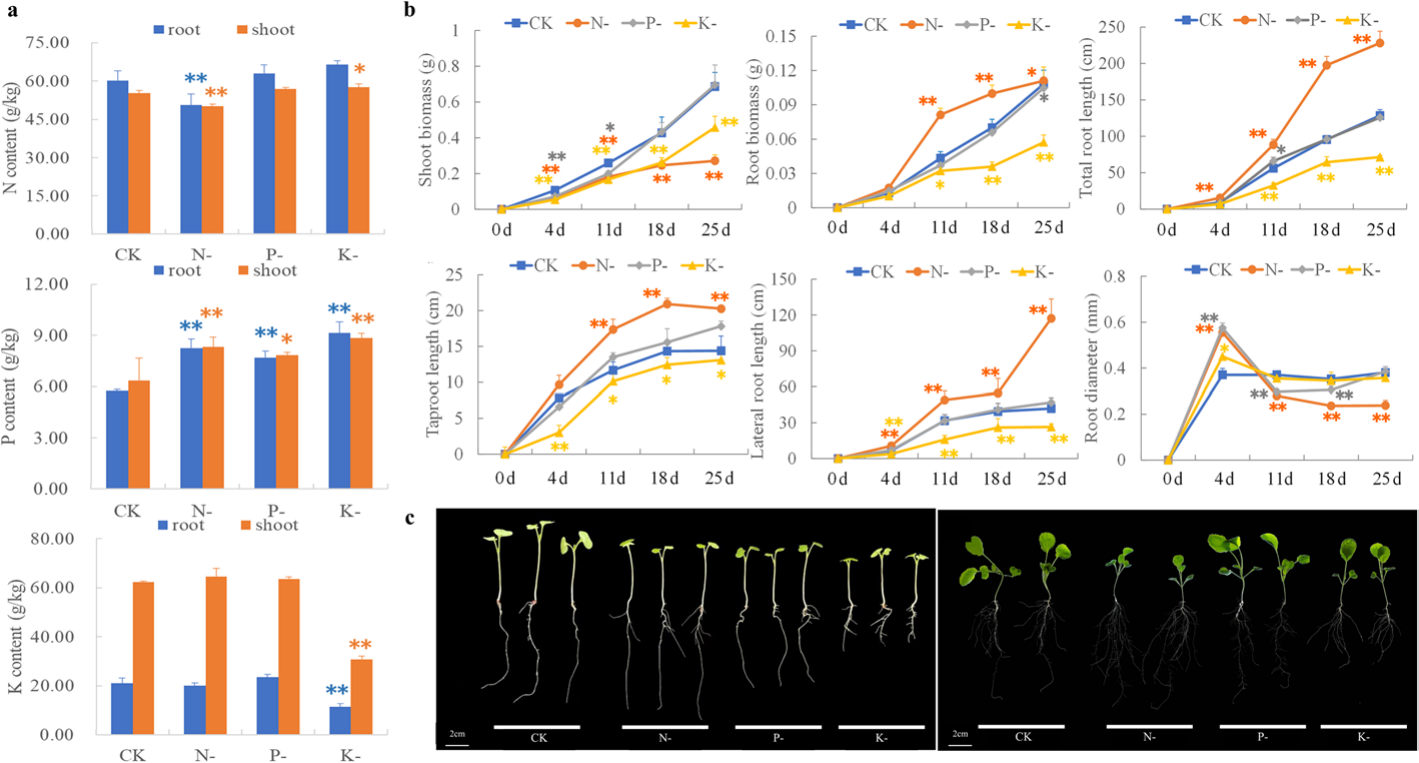
The phenotypic and physiological features of rapeseed seedlings exposed to N-, P- and K-deficiency stresses. **a** Plant macronutrient contents after 28 days of low-macronutrient treatments. **b** Morphological data, including root and shoot biomass, total root length, taproot length, lateral root length and root diameter, in different treatment periods. **c** Comparative growth performances of rapeseed seedlings after short-term (4 days) and long term (21 days) macronutrient deprivation treatments along with the full-nutrient control. Data are mean ± SD from three biological replicates. * and ** indicate a significant difference at *P* < 0.05 and *P* < 0.01 by the Tukey’s HSD test, respectively

Significant differences in final nutrient contents were observed among N, P, and K deprivation treatments. Unsurprisingly, lack of N supply resulted in significantly reduced shoot and root N contents (Fig. 1a), and K deprivation caused markedly decreased shoot and root K contents (Fig. 1a). However, significant increases in shoot and root P contents were observed not only after P withdrawal, but also after N and K withdrawal (Fig. 1a), suggesting a possible role of P availability in plant response to other macronutrient deficiencies.

The effects of macronutrient deficiency on biomass and shoot and root morphologies varied between N, P and K treatments. Obvious shoot growth inhibition was detected after N or K deprivation, while root growth was stimulated by N deprivation but inhibited by K deprivation (Fig. 1b, c). No obvious effects on shoot and root growth were observed under P deprivation compared with the control group, which corroborated the increased P content of seedlings observed under P deprivation stress.

**Fig. 2:**
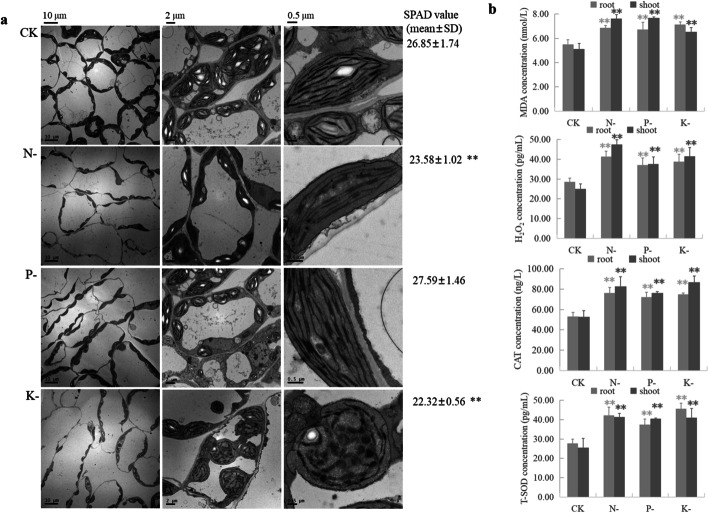
Chloroplast structure, chlorophyll content and reactive oxygen species accumulation in rapeseed seedlings after 28 days of low-macronutrient treatments along with a full-nutrient control. **a** Transmission electron microscopy (TEM) imaging of chloroplast structure and SPAD value that indicates relative chlorophyll content in the leaf tissue. **b** Reactive oxygen species levels in root and shoot samples after low-macronutrient treatments. Data are mean ± SD from three biological replicates. * and ** indicate a significant difference at *P* < 0.05 and *P* < 0.01 by the Tukey’s test, respectively

At 28 days following macronutrient withdrawal, the SPAD value (a measurement indicative of relative leaf chlorophyll content) decreased significantly in N- and K-deprived plants, but showed similar levels between P-deprived plants and controls (Fig. 2a). Observations of leaves by transmission electron microscopy (TEM) imaging revealed reduced chloroplast numbers, irregularly-shaped and rare thylakoid stacks in tiny layers of the stroma in N-deficient seedlings (Fig. 2a). K-deficient chloroplasts were more likely to be circular in shape rather than elliptical, and most chloroplasts showed disrupted grana structures, with swelling also observed. However, chloroplasts in P-deficient seedlings were similar to those of the controls. Disrupted chloroplast structure was synchronous with the loss of chlorophyll, leading to the loss of green color in N- and K-deficient leaves.

### ROS amounts are increased in N-, P- and K-deprived roots and shoots

Upon N, P and K macronutrient deprivations, malondialdehyde (MDA) and hydrogen peroxide (H_2_O_2_) concentrations in N-, P- and K-deficient roots and shoots increased significantly (Fig. 2b). Unsurprisingly, the concentration of enzymatic antioxidants responsible for scavenging excess accumulated ROS and alleviating oxidative injury were also increased under nutrient stress: catalase (CAT) contents were increased significantly in N-, P- and K-deprived roots and shoots, and total superoxide dismutase (T-SOD) were also increased significantly in roots and shoots subjected to N, P and K deprivations (Fig. 2b).

### Identification of specific commonalities of mRNA responses to N, P and K deprivation stresses

The sequence assembly results are summarized in Additional file 2: Table S1. RNA-seq profiling detected 5548, 4316 and 352 DEGs in N-, P- and K-deprived roots, and 637, 1174 and 349 DEGs in N-, P- and K-deprived shoots, with most DEGs being downregulated (Table 1, Additional file 2: Table S2). DEGs under N- and P-deficient conditions were far more numerous than DEGs under K-deficient conditions, and macronutrient-deprived roots generated more DEGs than shoots, suggesting higher plant sensitivity to external N or P status than K status, and greater sensitivity in roots than shoots.

**Table 1:** Numbers of differentially expressed mRNAs, miRNAs and circRNAs in *B. napus* roots and shoots under N, P and K deprivations

Type	Root			Shoot		
	N (up/down)	P (up/down)	K (up/down)	N (up/down)	P (up/down)	K (up/down)
mRNAs	5548 (2023/3525)	4316 (1768/2548)	352 (122/230)	637 (169/468)	1174 (176/998)	349 (148/201)
miRNAs	71 (33/38)	75 (30/45)	31 (13/18)	66 (32/34)	26 (5/21)	21 (12/9)
circRNAs	1 (0/1)	2 (0/2)	1 (0/1)	3 (0/3)	2 (2/0)	1 (1/0)
Metabolites	400 (129/271)	246 (91/155)	170 (78/92)	127 (67/60)	166 (37/129)	69 (56/13)

**Fig. 3:**
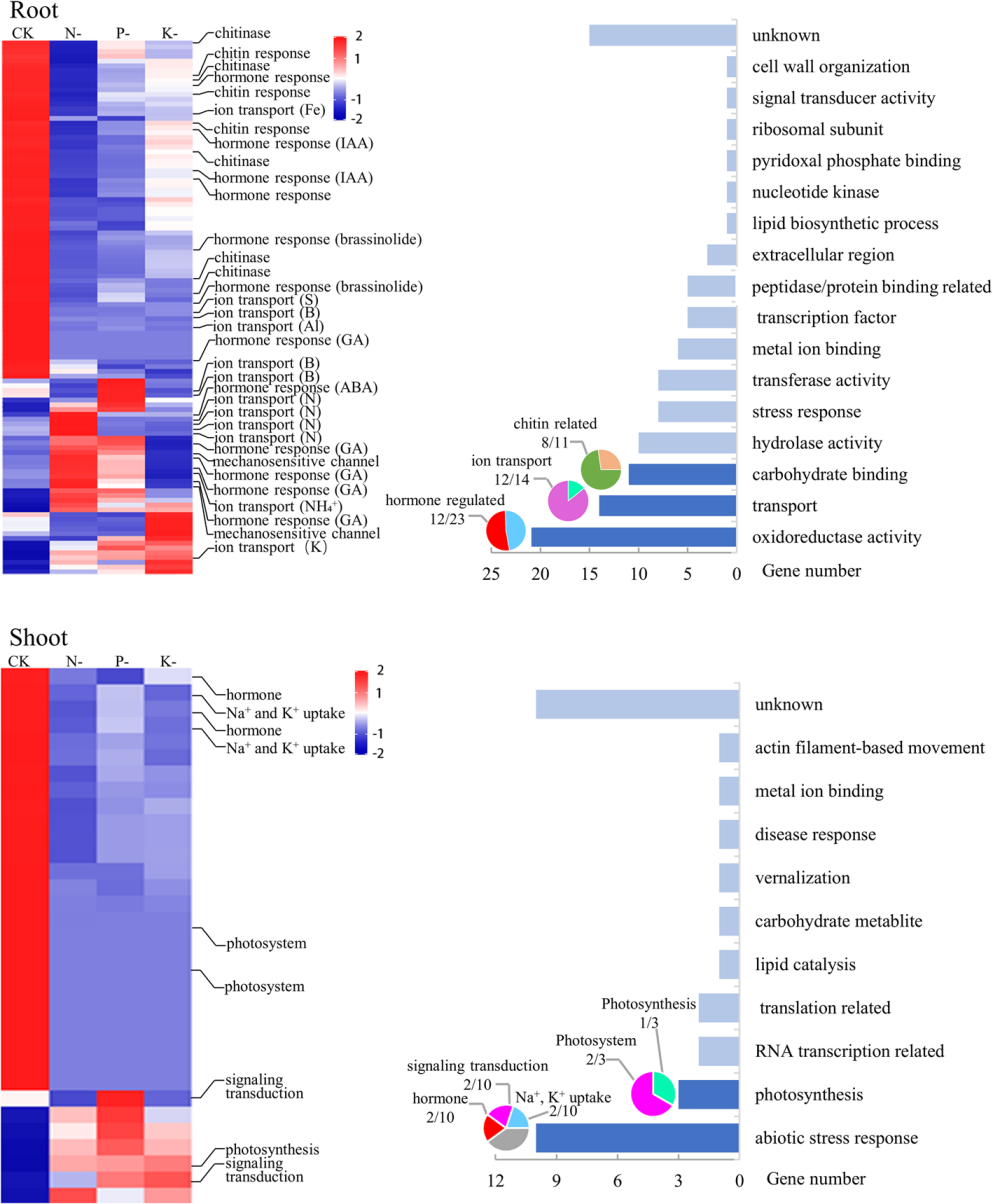
Global expression profiles (left) and functional classification (right) of 112 and 33 common differentially expressed genes (DEGs) in roots and shoots, respectively, of rapeseed seedlings after 28 days of N-, P- and K-deficiency treatments. The expression profiles were obtained based on log ratio (Log2 treatment/control) value. The functional classification was performed by gene ontology. The top three classes with the most genes were marked by pies and subgroups by gene annotations

A total of 112 DEGs in roots and 33 DEGs in shoots were identified across all macronutrient deficiency treatments, most of which were downregulated (Fig. 3). Of these DEGs, 87/112 in roots and 32/33 in shoots showed similar expression patterns under each of the different macronutrient deprivation stresses, suggesting that these DEGs may act as common response factors to macronutrient deprivation conditions. Of the common DEGs in roots, nearly half (48/112) were related to ‘oxidoreductase activity’, ‘transport’ and ‘carbohydrate binding’ (Fig. 3). Within these three groups, plant hormones (especially gibberellic acid biosynthesis), ion transport (especially nitrate/ammonium), and chitin-related genes (especially chitinase) were the most common components (Fig. 3). In shoots, the genes involved in ‘abiotic stress response’ and ‘photosynthesis’ accounted for 39% (13/33) of the common DEGs (Fig. 3). Of these, Na^+^ and K^+^ uptake, abscisic acid and ethylene response and plant signaling transduction accounted for 60% of the ‘abiotic stress response’ group, and the genes related to photosystem biogenesis accounted for 67% of the ‘photosynthesis’ group. These results suggest that common response factors across N, P and K macronutrient deprivation stresses in roots comprise genes with oxidoreductase activity, transport activity and carbohydrate binding activity, while common response factors in shoots comprise genes with signal transduction and photosynthesis activity.

### Oxidoreduction process and ion transmembrane transport are common responses mediated by miRNAs in response to different macronutrient deficiencies

A total of 71, 75 and 31 differentially expressed miRNAs (DEMs) were detected in N-, P- and K-deprived roots, and 66, 26 and 21 DEMs were identified in N-, P- and K-deficient shoots (Table 1, Additional file 2: Table S3). Of these, 10 miRNAs in roots and six miRNAs in shoots were identified as common response genes to each of N, P, and K deprivation stress, with their expression either upregulated or downregulated in both analyzed organs (Additional file 2: Table S3). Similar to mRNA data, the number of DEMs was much higher under N- and P-deprivation conditions than under K deprivation.

A total of 67, 73 and 17 DEMs under N-, P- and K-deprivation conditions in roots, and 49, 22 and 14 DEMs under N-, P- and K-deprivation conditions in shoots showed negative interactions with the target DEGs, suggesting putative miRNA regulation of these genes. Enriched biological processes for these DEGs are shown in Table 2: two processes, including ‘oxidatioN–reduction process’ and ‘ion transmembrane transport’, were identified under both N and P deprivation conditions in roots. Most enriched biological processes were specific to individual macronutrient deficiency stresses. For instance, N-deprivation in roots resulted in putative miRNA-regulated DEGs with enrichment in ‘cellular response to nitrogen starvation’, ‘folic acid-containing compound metabolism’ and ‘glycine biosynthesis’, P deprivation in roots resulted in putative miRNA-regulated DEGs with enrichment in ‘sterol biosynthesis’, ‘reactive oxygen species metabolism’ and ‘carbohydrate metabolism’, and K deprivation in roots resulted in putative miRNA-regulated DEGs with enrichment in ‘potassium ion transmembrane transport’ (Table 2). Fewer enriched biological processes in DEGs putatively regulated by miRNAs were found in the shoot tissue: only ‘reactive oxygen species metabolism’ and ‘negative regulation of cell death’ were affected under P deprivation conditions (Table 2).

**Table 2:** Enriched biological processes of target differentially expressed genes showing negative interactions with differentially expressed miRNAs in roots and shoots exposed to N-, P- and K-deprivation stresses in *Brassica napus*

GO_accession	Biological process	*P* value					
		N-R	P-R	K-R	N-S	P-S	K-S
GO:0055114	oxidatioN–reduction process	0.000	0.000	> 0.05	> 0.05	> 0.05	> 0.05
GO:0071577	Ion transmembrane transport	0.001	0.042	> 0.05	> 0.05	> 0.05	> 0.05
GO:0006995	Cellular response to nitrogen starvation	0.000	> 0.05	> 0.05	> 0.05	> 0.05	> 0.05
GO:0006760	Folic acid-containing compound metabolic process	0.001	> 0.05	> 0.05	> 0.05	> 0.05	> 0.05
GO:0006545	Glycine biosynthetic process	0.006	> 0.05	> 0.05	> 0.05	> 0.05	> 0.05
GO:0016126	Sterol biosynthetic process	> 0.05	0.007	> 0.05	> 0.05	> 0.05	> 0.05
GO:0072593	Reactive oxygen species metabolic process	> 0.05	0.018	> 0.05	> 0.05	0.000	> 0.05
GO:0005975	Carbohydrate metabolic process	> 0.05	0.021	> 0.05	> 0.05	> 0.05	> 0.05
GO:0071805	Potassium ion transmembrane transport	> 0.05	> 0.05	0.044	> 0.05	> 0.05	> 0.05
GO:0060548	Negative regulation of cell death	> 0.05	> 0.05	> 0.05	> 0.05	0.000	> 0.05

No DEG-miRNA target pairs were represented in all three macronutrient deficiency stresses, but a set of DEG-miRNA target pairs overlapped between two of the three macronutrient deficiency stresses (Additional file 1: Fig. S1). Under different macronutrient deficiency treatments, 27 common miRNA-mRNA pairs involving seven stress-responsive miRNA families (miR156, miR162, miR166, miR395, miR396, miR408 and miR4995) negatively interacted with 22 target DEGs which showed miRNA-mediated regulation of oxidoreduction processes. A further seven miRNA-mRNA pairs involving five differentially expressed miRNA families (miR156, miR157, miR162, miR397 and miR408) negatively interacted with seven target DEGs which played a role in ion transmembrane transport (Table 3). For oxidoreduction processes, the target genes mainly functioned as restorers of oxidative damage. These included ubiquitous enzymes, ascorbate peroxidases, and cytosolic copper/zinc superoxide dismutase. Almost all of the target genes were down-regulated by up-regulated miRNAs under macronutrient deficiency conditions. By contrast, for transmembrane transport, miRNA-mediated transporters involved both up-regulated and down-regulated transporters, including up-regulation of *monosaccharides transporters* (*ESL1*), *K* + *transporter 5* (*HAK5*) and *phosphate transporter 1;3* (*PHT1;3*), and down-regulation of *Fe(II) transporter* (*ZIP9*), *cyclic nucleotide-binding transporter 1* (*CNGC20*) and *zinc transporter 4* (*ZIP4*).

**Table 3:** The common miRNA-target gene pairs involved in oxidatioN–reduction/oxidative stress response and transmembrane transport in response to different macronutrient stress in *B. napus*

GO annotation	Treatment-tissue	miRNAs		Targets		Gene name	Target function
		ID	Regulation	ID	Regulation		
oxidatioN–reduction/oxidative stress response	N-R, P-R	cpa-miR166e	Up	BnaA02g37030D	Down	*SQE5*	Squalene monooxygenase
	N-R, P-R	tae-miR395b	Up	BnaA03g02060D	Down	*PMSR3*	Ubiquitous enzyme
	N-R, P-R	tae-miR395b	Up	BnaA03g02070D	Down	*PMSR3*	Ubiquitous enzyme
	N-R, P-R	tae-miR395b	Up	BnaA03g47130D	Down	*RBOHG*	Riboflavin synthase-like superfamily protein
	N-R, P-R	gma-miR166u	Up	BnaA03g56840D	Down	*CYP704A2*	Incorporation or reduction of molecular oxygen
	N-R, P-R	zma-miR156k-5p	Up	BnaA06g05150D	Down	*SOD1*	Cytosolic copper/zinc superoxide dismutase CSD1
	N-R, P-R	vvi-miR396b	Up	BnaA08g16100D	Down	*CYP79B2*	Tryptophan metabolism
	N-R, P-R	zma-miR156k-5p	Up	BnaA09g49190D	Down	*APX1*	Ascorbate peroxidases
	N-R, P-R	ath-miR396b-5p	Up	BnaAnng01300D	Down	*PRX52*	Peroxidases
	N-R, P-R	vvi-miR396b	Up	BnaAnng01300D	Down	*PRX52*	Peroxidases
	N-R, P-R	vvi-miR396b	Up	BnaAnng38110D	Down	*-*	Flavanone 3 hydroxylase
	N-R, P-R	ath-miR396b-5p	Up	BnaC02g02350D	Down	*PRX52*	Peroxidases
	N-R, P-R	vvi-miR396b	Up	BnaC02g02350D	Down	*PRX52*	Peroxidases
	N-R, P-R	aly-miR408-5p	Down	BnaC02g18050D	Up	*NDHG*	NADH dehydrogenase ND6
	N-R, P-R	zma-miR156k-5p	Up	BnaC02g33320D	Down	/	Auxin-responsive family protein
	N-R, P-R	cpa-miR166e	Up	BnaC02g41390D	Down	*SQE5*	Squalene monooxygenase gene homolog
	N-R, P-R	tae-miR395b	Up	BnaC03g03070D	Down	*PMSR3*	Ubiquitous enzyme that repairs oxidatively damaged proteins
	N-R, P-R	tae-miR395b	Up	BnaC03g03110D	Down	*PMSR3*	Ubiquitous enzyme that repairs oxidatively damaged proteins
	N-R, P-R	vvi-miR396b	Up	BnaC03g60820D	Down	*CYP79B2*	Tryptophan metabolism
	N-R, P-R	ath-miR396b-5p	Up	BnaA09g28900D	Down	*BBE7*	FAD-binding Berberine family protein
	N-R, P-R	vvi-miR396b	Up	BnaA09g28900D	Down	*BBE7*	FAD-binding Berberine family protein
	N-R, P-R	ath-miR396b-5p	Up	BnaC05g20360D	Down	*BBE7*	FAD-binding Berberine family protein
	N-R, P-R	vvi-miR396b	Up	BnaC05g20360D	Down	*BBE7*	FAD-binding Berberine family protein
	N-R, P-R	zma-miR162-3p	Up	BnaC07g36420D	Down	*BBE20*	Oligogalacturonide oxidase
	N-R, P-R	zma-miR166h-3p	Up	BnaCnng59310D	Down	*BBE8*	FAD-binding Berberine family protein
	N-S, K-S	gma-miR4995	Up	BnaA02g07630D	Down	/	Peroxidase superfamily protein
Transmembrane transport	N-R, P-R	ath-miR408-5p	Down	BnaA06g05200D	Up	*ESL1*	A transporter for monosaccharides
	N-R, P-R	ath-miR397a	Down	BnaA07g16500D	Up	*HAK5*	KUP/HAK/KT potassium channel
	N-R, P-R	ath-miR397a	Down	BnaA09g16490D	Up	*PHT1;3*	Phosphate transporters
	N-R, P-R	zma-miR156k-5p	Up	BnaC01g05220D	Down	*ZIP9*	Fe(II) transporter isolog
	N-R, P-R	zma-miR162-3p	Up	BnaC03g40070D	Down	*CNGC20*	Cyclic nucleotide-binding transporter 1
	N-P, K-P	ath-miR157a-3p	Up	BnaA09g47880D	Down	*ZIP4*	Zrt- and Irt-related protein (ZIP) family
	N-P, K-P	ath-miR157a-3p	Up	BnaC08g42250D	Down	*ZIP4*	Zrt- and Irt-related protein (ZIP) family

### circRNAs play minor roles in response to N-, P- and K-deprivation stresses

**Fig. 4:**
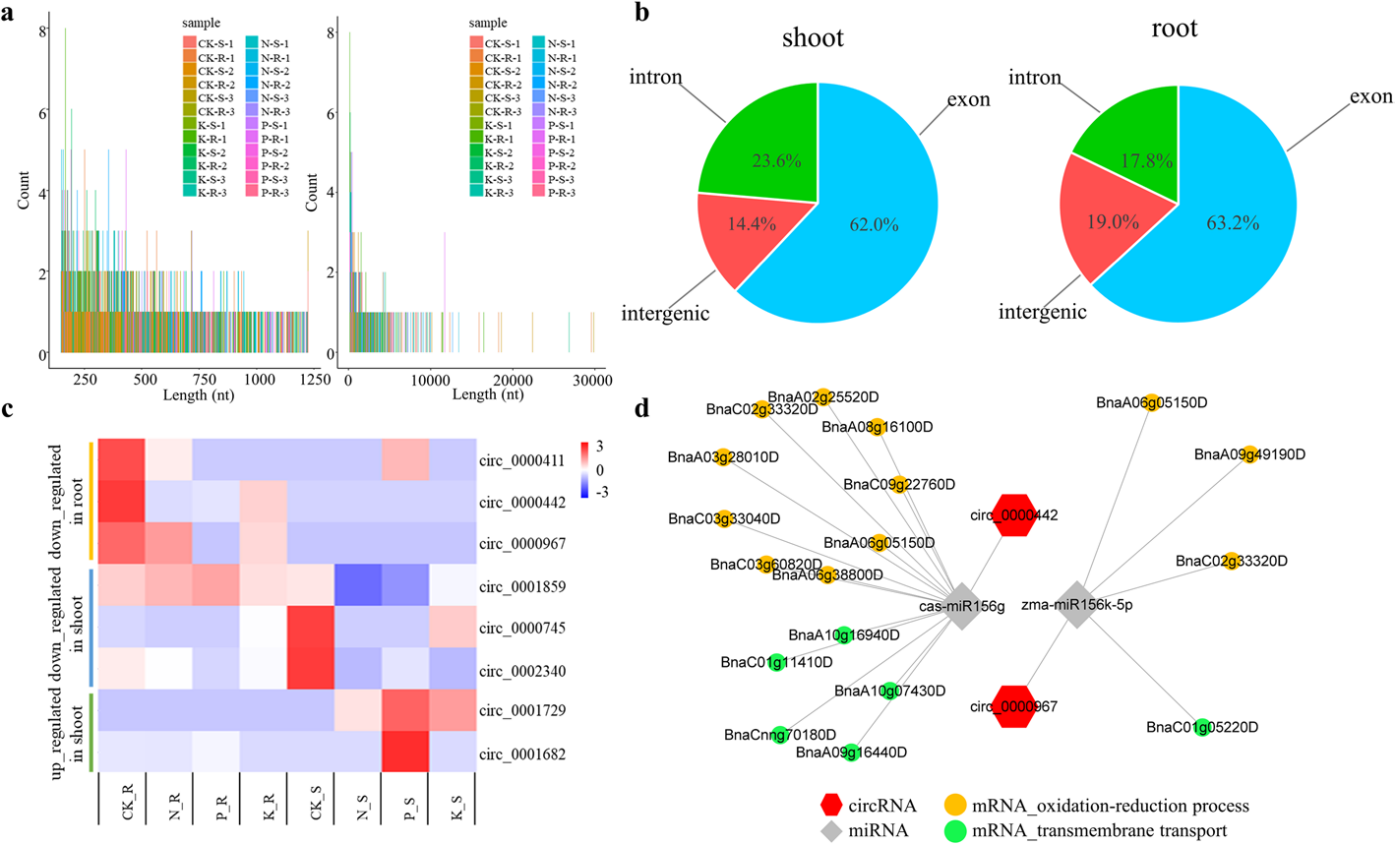
Identified circRNAs in rapeseed seedlings after 28 days of N-, P- and K-deficiency treatments. **a** Length distributions of identified circRNAs. **b** Proportions of exonic circRNAs, intronic circRNAs and intergenic circRNAs. **c** Expression profiles of differentially expressed circRNAs. **d** Differentially expressed circRNA–miRNA–mRNA co-expression networks

In total 2620 candidate circRNAs were identified. The lengths of the identified circRNAs varied across a wide range (Fig. 4a), indicating that there may be more than one binding site and multiple RNA binding proteins per circRNA. 63.0% of the circRNAs were located in exons, 18.5% were located in introns and 18.5% were intergenic (Fig. 4b).

Fewer differentially expressed circRNAs were detected in response to macronutrient deficiency than differentially expressed mRNAs and microRNAs. One upregulated and four downregulated circRNAs were detected in roots, versus one upregulated and three downregulated circRNAs in shoots (Additional file 2: Table S4) (Fig. 4c). Of these, two circRNAs were differentially expressed under different macronutrient deficiencies: circ_0000442 was differentially expressed in both N- and P-deprived roots, and circ_0001729 was differentially expressed in both P- and K-deprived shoots.

In order to determine if circRNAs were functioning as decoys for miRNA, circRNA–miRNA–mRNA networks were constructed. As shown in Fig. 4d, there were 18 putative interactions between two circRNAs, two miRNAs, and 16 mRNAs in N- and P-deprived roots, where circRNAs might function as miRNA sponges under N- and P-stress conditions in rapeseed. Gene annotation analysis of these correlated mRNAs suggested that these two circRNAs act as decoys for miR156 and participate in oxidoreduction processes and transmembrane transport in both N- and P-deprived roots (Fig. 4d, Additional file 2: Table S4). Of the genes involved in oxidoreduction processes, plant hormone-related processes were predominant: auxin and brassinosteroid biosynthesis and responses were involved in circRNA-mediated root response to N deprivation, while auxin and jasmonic acid responses were involved in circRNA-mediated root response to P deprivation. In summary, circRNAs may play only a minor role in macronutrient deficiency induced response: only two stress-responsive circRNAs were identified, which interacted with two miRNAs and 16 mRNAs downstream, putatively participating in oxidoreduction processes and transmembrane transport.

### Dramatic metabolite alterations occur in response to N, P and K deprivation treatments

An untargeted metabolomic approach was subsequently utilized to detect the effects of different macronutrient deficiencies on plant metabolites. More differentially abundant metabolites (DAMs) were detected in roots, with 400, 522, and 318 DAMs under N-, P- and K-deprivation conditions, respectively, compared with shoots, with 268, 370 and 135 DAMs under N-, P- and K-deprivation conditions, respectively (Table 1), which corroborates with the observation of higher numbers of DEGs and DEMs in roots than in shoots.

DAMs were grouped into ten classes according to functional annotation (Table 4). In N-, P- and K-deprived roots, most DAMs showed reduced abundance, with heterocyclic compounds and alkaloids showing the most substantial changes under all three macronutrient-deprivation stresses. Amino acids were also changed markedly, mostly decreasing in abundance under different macronutrient deficiencies. These results are consistent with a possible adaptive mechanism in plants to turn off or reduce root metabolite production when faced with nutrient-limiting conditions, which is also consistent with the high proportions of downregulated mRNAs, miRNAs and circRNAs observed in the root tissue. Interestingly, several metabolite groups were also produced in higher levels under macronutrient stress conditions. In particular, lipids and lipid-like components were produced at higher levels in both roots and shoots under N- and K-deprivation conditions (Table 4). Unlike roots, which showed mostly decreased metabolite levels, shoots tended to have increased metabolite production under nutrient stress. This was observed for aldehydes, ketones and quinones, carbohydrates, heterocyclic compounds and alkaloids in N- and K-deprived shoots, suggesting a positive role for these metabolites in plant response to macronutrient deficiency stresses.

**Table 4:** Classification of differentially abundant metabolites in the roots and shoots of *Brassica napus* exposed to N, P and K deprivations

Tissue	Classification	N-			P-			K-		
		Total	Up	Down	Total	Up	Down	Total	Up	Down
Root	Amino acids	27	3	24	66	8	58	12	1	11
	Alcohol, ether and phenol	24	9	15	33	12	21	10	4	6
	Aldehydes, ketones and quinones	38	10	28	29	9	20	26	6	20
	Carbohydrate	25	11	14	33	20	13	27	7	20
	Carboxylic acids and derivatives	38	9	29	45	12	33	19	3	16
	Heterocyclic compounds and alkaloids	79	18	61	98	23	75	57	13	44
	Hydrocarbons	29	12	17	27	9	18	15	7	8
	Lipids and lipid-like	46	32	14	75	16	59	68	51	17
	Nucleosides and nucleotides	12	0	12	18	4	14	10	6	4
	Organic nitrogen compounds	8	4	4	9	6	3	11	3	8
	Unclassified	74	21	53	89	25	64	63	28	35
Shoot	Amino acids	31	12	19	38	4	34	16	14	2
	Alcohol, ether and phenol	9	6	3	11	1	10	2	1	1
	Aldehydes, ketones and quinones	22	15	7	16	6	10	11	7	4
	Carbohydrate	29	15	14	17	8	9	15	11	4
	Carboxylic acids and derivatives	18	12	6	33	17	16	10	8	2
	Heterocyclic compounds and alkaloids	45	25	20	84	23	61	28	25	3
	Hydrocarbons	13	7	6	16	3	13	0	0	0
	Lipids and lipid-like	33	27	6	84	13	71	24	17	7
	Nucleosides and nucleotides	12	4	8	15	4	11	3	2	1
	Organic nitrogen compounds	7	1	6	3	1	2	6	5	1
	Unclassified	49	23	26	53	16	37	20	13	7

Several plant hormones showed significant responses to N-, P- and K-deficiency conditions: abscisic acid (ABA), auxin compounds (indole-3-acetic acid, IAA; indole-3-carboxaldehyde, ICA; indole-3-carboxylic acid, ICAA; methyl indole-3-acetate, ME-IAA), jasmonic acid compounds (methyl jasmonate, MeJA), and cytokinin compounds (trans-zeatin, tZ; trans-zeatin-riboside, tZR). Plant hormone changes were more common in shoots than in roots. Several plant hormones were found to respond to all three macronutrient deprivation stresses. For example, the auxin compounds ICA and ICAA were significantly increased in both N- and P-deprived shoots and roots, while the cytokinin compound tZR was significantly increased across all three macronutrient-deficiency conditions in roots, and the jasmonic acid compound MeJA was reduced in roots under all three macronutrient-deficiency stresses.

### Several key metabolites involved in the adaptation to macronutrient deficiency were identified by meta-analysis of RNAs and metabolites

Several enriched biological processes detected by RNA-sequencing were validated by observations of differential downstream metabolite production (Table 5). Of these, enhanced accumulation of tricarboxylic acid, oxazole or thiazole and carbohydrates, and catabolism of aromatic amino acid and glucosamine-containing compounds showed consistent RNA-to-metabolite changes in both N- and P-deprived roots. Enhanced sterol biosynthesis and corresponding increases in sterol metabolites were also found in P- and K-deprived roots. In shoots, auxin response was detected in both RNAs and metabolites under each of N-, P- and K-deprivation conditions. Accumulation of cinnamic acid, nicotianamine and glutamine family amino acids was also reduced based on RNA and metabolite results in N-deprived roots, while aspartate amino acid metabolism was repressed in N-deprived shoots. The combined analysis of enriched biological processes from RNA-sequencing and differentially abundant metabolites suggested positive roles for tricarboxylic acid, azole, carbohydrate, sterol and auxins, and negative roles for aromatic and aspartate amino acids, glucosamine-containing compounds, cinnamic acid, and nicotianamine in plant adaptation to macronutrient deficiency stress.

**Table 5:** Comparative analysis between enriched biological processes from RNA sequencing and differentially abundant metabolites in *Brassica napus* under N, P and K deprivations

Sample^a^	Transcriptome	Metabolites	
	Enriched biological processes	Up	Down
N-R	Aromatic amino acid family catabolism*	-	D-( +)-Tryptophan
	Tricarboxylic acid biosynthesis*	DL-Malic acid; Succinic acid	-
	Oxazole or thiazole biosynthesis*	Epigoitrin	-
	Carbohydrate derivative catabolism	Nystose; D-( +)-Maltose; D-Gluconic acid	Isomaltose; Acetaminophen glucuronide; Mucic acid
	Cinnamic acid biosynthesis	-	Trans-cinnamic acid; Methyl cinnamate; Cinnamic Acid
	Glucosamine-containing compound metabolism	-	UDP-N-acetylglucosamine
	Glutamine family amino acid metabolism	-	DL-Glutamine; D-(-)-Glutamine
	Nicotianamine metabolism	-	Nicotianamine
P-R	Aromatic amino acid family catabolism*	-	Alanyltyrosine; gamma-Glutamyltyrosine; L-Tyrosine; L-Phenylalanine; N-Acetylphenylalanine
	Tricarboxylic acid biosynthesis*	Methylenesuccinic acid; 2,2-Dimethylsuccinic acid; 2-Isopropylmalic acid	Citric acid; D-threo-Isocitric acid
	Oxazole or thiazole biosynthesis*	2-Mercaptobenzothiazole	N-(4-methoxy-1-methyl-1H-indazol-3-yl)-5-methyl-3-isoxazolecarboxamide
	Sterol biosynthesis*	Estrone sulfate	-
	Aminoglycan catabolism	-	Glucosamine
P-R	Tryptophan catabolism	5-Hydroxytryptophan	-
	Disaccharide biosynthesis	Sucrose; D-( +)-Maltose	-
	Oligosaccharide biosynthesis	Stachyose; D-Raffinose; Nystose	-
	Fructose 6-phosphate metabolism	D-Mannose 6-phosphate	-
	Trehalose biosynthesis	α,α-Trehalose	-
	Indole-containing compound catabolism	Indole-3-carboxylic acid; 3-Indolepropionic acid	6-Hydroxymelatonin
K-R	*Sterol biosynthesis	Stigmasterol; Vitamin D2; Ergocalciferol	-
N-S	Response to auxin stimulus	Indole-3-carboxaldehyde	-
	Response to hormone stimulus	Indole-3-carboxaldehyde; Abscisic Acid	Gibberellin A4
	Aspartate family amino acid metabolism	-	Asparagine
P-S	Indole-containing compound catabolism	Tryptophol; Methyl indole-3-acetate; Indole-3-carboxaldehyde	Indole-3-acetic acid; Indole-3-carboxylic acid; 3-Indoleacetonitrile; L-Tryptophan; 2-(1H-indol-3-yl)acetic acid; Indole
	Tryptophan catabolism		L-Tryptophan
	Aromatic compound biosynthesis	2-Hydroxyphenylalanine; N-Acetylphenylalanine; L-Tryptophan	-
K-S	Response to auxin stimulus	Indole-3-acetic acid; Indole-3-acetamide; 3-Indolepropionic acid	-

^a^*N-R* root tissue under N deficiency stress; *P-R* root tissue under P deficiency stress; *K-R* root tissue under K deficiency stress; *N-S* shoot tissue under N deficiency stress; *P-S* shoot tissue under P deficiency stress; *K-S* shoot tissue under K deficiency stress

*Enriched biological processes responsive to different macronutrient-deficiency stresses

### Validation of differentially expressed RNAs by qRT-PCR

To validate the credibility of the RNA-seq data, we subjected twelve differentially expressed mRNAs, miRNAs and circRNAs to qRT-PCR. Eleven of the twelve RNAs showed the same expression changes between the RNA-seq and qRT-PCR results (Additional file 1: Fig. S2), with correlation coefficients ranging from 0.80 to 1.00 (Additional file 1: Fig. S2). Only one RNA, miRNA bra-miR2111b-3p, showed inconsistent expression patterns between RNA-seq and qRT-PCR. Comparisons of gene expression results between methods often show a small but specific gene set with inconsistent expression measurements: these genes tend to have smaller gene sequences, fewer exons, and lower expression levels, e.g. miRNAs [33]. The high correlation between the two methods validated the sequencing results, indicating that RNA-seq accurately assessed and identified genes involved in macronutrient deficiency stresses in *B. napus*.

## Discussion

We investigated differential expression of mRNAs, miRNAs, circRNAs and metabolites in roots and shoots concurrently under different macronutrient stresses. Macronutrient stress alters normal plant growth and development through direct induction of nutrient imbalances, ion damage, and ROS production. Consequently, the plant response to macronutrient stress involves various physiological and biochemical events initiated by multiple genes. Interestingly, we found that macronutrient deficiency had the greatest impact on miRNAs (152 differentially expressed miRNAs out of a total of 549 miRNAs identified), followed by metabolites and mRNAs (9385 differentially expressed genes out of a total of 102,278 genes identified), and that macronutrient deficiency affected circRNAs only rarely (8 differentially expressed circRNAs out of a total of 2620 circRNAs identified). These results corroborate the important role of miRNAs in regulating plant adaptive responses under different stress conditions. Although circRNAs are known to contribute to adaptive responses to abiotic stresses (low temperature stress, salt stress, and drought stress) [27–29], our results suggest that circRNAs are not as affected as miRNAs by macronutrient deprivation stress in rapeseed.

Surprisingly, we observed no obvious differences in either roots or shoots following 28 days of P deprivation. A short-term decrease in root and shoot growth was observed, but this was compensated for before the end of the experiment (Fig. 1b, c). P is a structural constituent of nucleic acids, membrane lipids, and energy metabolites, and has important functions in signal transduction. For the majority of plants, low P usually inhibits primary root growth, enhances lateral root formation, and suppresses shoot growth. The lack of observable morphological differences in this study was also coupled with significantly increased plant P content under P deprivation (Fig. 1a). These results might suggest highly efficient P absorption in the rapeseed genotype used in the present study, or other adaptive strategies that can alleviate or otherwise help with toleration of P deficiency. Our gene expression analyses validated this notion: 229 genes related to transport processes were identified as up-regulated in response to P deficiency, including six phosphate transporters which would potentially enhance P absorption (Additional file 2: Table S5). These comprised four copies of *early-responsive to dehydration stress* (*ERD4*), which are involved in the activity of calcium permeable stress-gated cation channel 1 and function as putative phosphate transporters, one gene named *glycerol-3-phosphate permease 2 (G3PP2)*, which has been confirmed to be a functional vacuolar P exporter for its homologous genes *OsVPE1* and *OsVPE2* in *O. sativa* [34], and one gene named *phosphate transporter-1;3* (*PHT1;3*), which mediates P uptake, translocation, and remobilization [35]. A set of more general phosphate starvation responders was also identified, including *purple acid phosphatase* genes (*PAPs*). Additionally, zinc ion transmembrane transport processes were significantly enriched in P-deprived roots: for example, a set of zinc/iron permease genes altered expression upon P deprivation (Additional file 2: Table S2). These processes may exert specific effects in signaling pathways that regulate high-affinity P transporter genes, subsequently increasing P accumulation [36].

Our results suggest that N and K deficiencies may also regulate plant P responses. Coordinated acquisition of different mineral elements has been reported to be critical for plants to achieve nutritional balance in environments with fluctuating nutrient availability [37]. As shown above, N limitation significantly enhanced P accumulation in both roots and shoots, suggesting an interplay between N and P. N was previously shown to act as a signaling molecule for modulating phosphate response and coordinating the N-P balance: genes such as *nitrate transporter* (*NRT1.5*), *SPX domain gene 4* (*SPX4*), *phosphate transporter1;1* (*PHT1;1*), *phosphate transporter1;4* (*PHT1;4*), and *nitrate-inducible GARP-type translational repressor1.4* (*NIGT1.4*) have been implicated in the N-P interplay [37, 38]. In this study, ten phosphate transporters were upregulated in the roots under N deprivation, which could explain the increased P content observed: putatively, the N deprivation-induced P response involves accelerating P transport. Similar to N-regulated P increase, K deprivation also resulted in increased P content following the upregulation of a set of P deprivation-induced genes. These included three genes encoding phosphoethanolamine/phosphocholine phosphatase, which is involved in the liberation of inorganic phosphate from intracellular sources, and two phosphate deprivation-induced genes encoding pyrophosphate-specific phosphatase. However, K deprivation did not affect the expression of P transporters. Although a K-P interaction has not been widely reported so far, our study suggests a K-regulated P response and highlights possible mechanisms for both N-P and K-P interplays. The molecular mechanisms underlying N-P interactions and how K deprivation triggers phosphate deprivation signals are interesting topics for future investigation.

An integrative overview of the responses of a given plant species to different nutrient deprivation conditions are essential for a better understanding of plant adaptive mechanisms. This is especially necessary for crop species with high nutrient (fertilizer) demands such as *Brassica napus* [39]. However, to date few studies have compared response of *B. napus* to all three macronutrients deprivations separately under the same experimental conditions [40]. To the best of our knowledge, only one previous study has examined multiple macronutrient deprivation conditions using transcriptomics: Courbet et al. (2021) assessed the impact of reduced availability of N, Mg, P, S, K, and Ca in root tissue [40]. In this study, 18 common DEGs were found between N, P, and K deprivation treatments (relative to 112 common DEGs detected in our study). Similar to our results, transport processes were found to be enriched under all macronutrient deprivation conditions. Although none of genes identified by as common DEGs under N, P, and K deprivation overlapped between the two studies, it is worth noting that sulphate anion transporters were identified as common response factors to N, P, and K deprivation in both studies (encoded by *BnaC03g09670D* and by *BnaC02g14670D*). These results support a previous hypothesis of crosstalk between sulfur and macronutrient availability of N, P and K [41], giving credence to the relative importance of sulphate anion transporters for plant adaption to a range of macronutrient availability conditions.

In plants, miRNAs have previously been revealed to participate in plant adaptation to macronutrient deficiency [42]. However, to date, no research has been carried out in rapeseed to compare miRNA-triggered responses between different nutrient deprivation conditions. In the present study, we found miRNA-mediated regulation of oxidatioN–reduction/oxidative stress response and transmembrane transport in response to different macronutrient stresses. In particular, several common miRNA-mRNA gene pairs were identified as candidates for macronutrient response in rapeseed. Seven miRNA families (miR156, miR162, miR166, miR395, miR396, miR408 and miR4995) were implicated as having a regulatory role in oxidatioN–reduction/oxidative stress response and five miRNA families (miR156, miR157, miR162, miR397 and miR408) were implicated as having a regulatory role in transmembrane transport (Table 3). Specifically, we found that lack of macronutrients up-regulated expression of miR156, miR157 and miR162, and subsequently suppressed a set of transporter targets: *Fe(II) transporter* (*ZIP9*), *cyclic nucleotide-binding transporter 1* (*CNGC20*) and *zinc transporter 4* (*ZIP4*)*.* In contrast, the expression of miR397 and miR408 was down-regulated in response to N or P deficiency, stimulating the expression of transporters such as *monosaccharides transporters* (*ESL1*), *K* + *transporter 5* (*HAK5*) and *phosphate transporter 1;3* (*PHT1;3*). In plants, miR397 and miR408 were first identified to show decreased expression upon N limitation in Arabidopsis, while miR156 was found to be up-regulated under N, P or S deprivation conditions in *Camellia sinensis*, *Lupinus angustifolius* and *Brassica napus* [43–45], and miR162 was found to be up-regulated under N or Fe deprivation conditions in rice and maize [46, 47]. These results are consistent with our observations of decreased expression of miR397 and miR408 and increased expression of miR156 and miR162 in response to N or P deficiency in our study. Hence, miR397, miR408, miR156 and miR162 may comprise major players in maintenance of multi-nutrient homeostasis.

OxidatioN–reduction and reactive oxygen species homeostasis play important roles in maintaining cellular tolerance to stress. With respect to oxidatioN–reduction/oxidative process, we observed a total of seven putatively regulatory miRNA families (miR156, miR162, miR166, miR395, miR396, miR408 and miR4995) forming 26 miRNA-mRNA gene pairs showing responses under different macronutrient deprivation conditions. Six of these seven miRNA families (all except miR4995) were previously identified as responsive to at least one different nutrient deprivation condition in other species [48]. In our study, we found that each of these miRNAs responded via upregulation to at least two different macronutrient deficiencies (except for miR408 which was down-regulated), and subsequently suppressed the corresponding gene targets. The expression patterns of some miRNA-mRNA gene pairs in our study were in contrast to the published results: for example, N-deficient maize plants were found to downregulate miR166, while K deficiency resulted in down-regulation of miR395, but both were instead upregulated in rapeseed in our study. Contrasting expression patterns of miRNA in response to nutrient deficiency have been described previously, and have been attributed to differences between species, genotypes, growing conditions, and treatment procedures: for example, miR159 was down-regulated by P limitation in *Betula luminifera* but up-regulated by N limitation in *Cucumis sativus* [48]. In summary, we found seven oxidatioN–reduction genes which were (mostly) down-regulated by up-regulation of miRNAs under at least two different macronutrient deprivation conditions, supporting a common mechanism for plant adaptation to reduced nutrient availability.

**Fig. 5:**
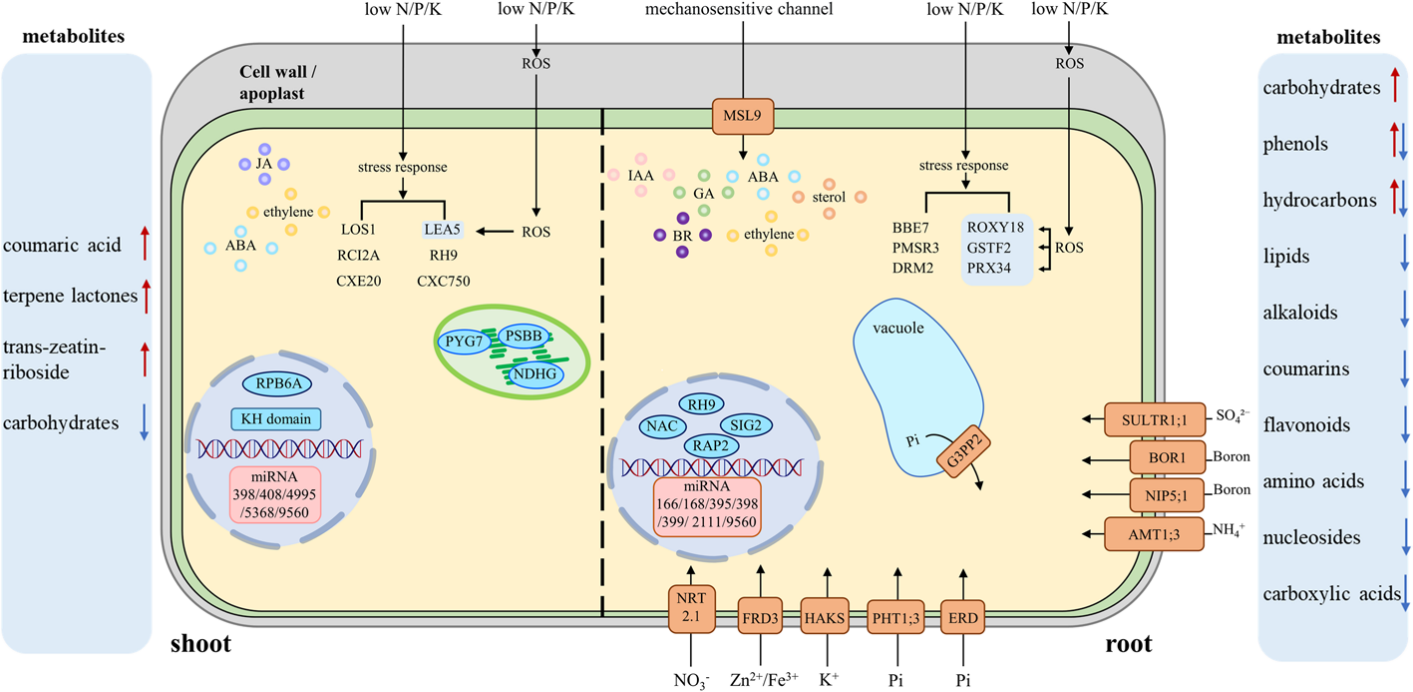
A schematic model of common factors induced by N, P and K deficiencies in rapeseed

Based on a comparative analysis in this study, we identified several mRNAs, miRNAs and metabolites as common response factors in rapeseed under N, P and K limitation (Fig. 5). Genes involved in common responses to N, P and K deficiencies could be classified into seven functional groups: ion transporters, molecular signal transduction, ROS scavengers, stress responders, miRNAs, transcription factors and photosystem biogenesis/photosynthesis-related genes. Three common factors involving photosystem biogenesis/photosynthesis were specifically identified in the shoot, suggesting impaired photosynthesis under N-, P- or K-deprivation conditions. In root and shoot tissues, several genes related to signal transduction pathways (such as IAA, ethylene, ABA, BR and GA, along with sterols), were potentially involved in local and systemic sensing and signaling pathways which were regulated by deprivation of each of the three macronutrients. This is consistent with results from previous studies, which identified phytohormones auxin, ethylene, cytokinin, gibberellins and abscisic acid to modulate gene response and subsequently root structure under N or P deficiency [49–51]. Several ROS scavengers, stress-responsive genes, transcription regulators and miRNAs were also over-represented under N-, P- and K-deficiency stress conditions in both root and shoot. Ion transporters were also identified in the root and photosystem biogenesis/photosynthesis-related genes identified in the shoot. Ion transporters are known to be involved not only in transport of macronutrients (N, P and K), but also transport of other elements such as sulphur, boron, zinc and iron. Hence, the common involvement and regulation of ion transporters might provide an explanation for crosstalks between nutrients, and a mechanism by which deficiency of one macronutrient might alter the uptake of another element [49].

Metabolic regulation is a key strategy for plant adaption to various stresses. Although metabolomic responses of plants to abiotic stresses have been examined by multiple studies, the latter were limited to one or at most two abiotic stressors. In the present work, 48 metabolites in the root and eight metabolites in the shoot were found to be responsive to all three macronutrient-deficiency stresses. Of these, sugars such as nystose and maltose were accumulated in root tissue under all three macronutrient-deficiency stresses, which was consistent with previous studies and validated the notion that sugars are beneficial for the enhancement of abiotic stress tolerance [52]. The phytohormone cytokinin-derivative trans-zeatin-riboside also accumulated in the shoot under all three macronutrient-deficiency stresses, suggesting an important function of this cytokinin in macronutrient response and corroborating the previously reported roles of cytokinins in response to abiotic stressors such as temperature, drought, osmotic pressure, salt, and nutrient stress [53].

## Conclusions

Exploring the mechanisms underlying common plant responses to different macronutrient deficiencies is important to facilitate plant breeding goals related to nutrient use efficiency, and hence for the subsequent production of crops which maintain normal growth and development in multiple-macronutrient-limited environments. However, few studies to date have compared N-, P- and K-nutrient deficiencies to identify common responsive factors. The investigation of synchronous regulation of mRNAs, miRNAs, circRNAs and metabolites under N-, P- and K-nutrient deprivation stresses in this study provided important insights into the regulatory network involved in the interaction between multiple nutrients, which could be important for breeding resilient crop plants. In summary, we assessed phenotypic and physiological changes in rapeseed in response to deprivation of macronutrients N, P and K, and identified mRNAs, miRNAs, circRNAs and metabolites involved in common and specific plant responses. Associations of RNAs with metabolites in plant adaptation to macronutrient deficiency were also assessed. We identified genes and processes in rapeseed which can be targeted to reduce macronutrient deficiency stress: putative strategies suggested include reducing the expression of non-essential genes, and activating or enhancing the expression of anti-stress genes, aided by plant hormones and ion transporters. The identification of common responders to different macronutrient deficiencies has importance in guiding breeding for enhancing nutrient use efficiency in rapeseed.

### Supplementary Information

**Additional file 1.** Supplementary Figures.

**Additional file 2.** Supplementary Tables.

## Abbreviations

N: Nitrogen
P: Phosphorus
K: Potassium
ROS: Reactive oxygen species
miRNA: MicroRNA
circRNAs: Circular RNA
DEG: Differentially expressed gene
DEM: Differentially expressed miRNA
DEC: Differentially expressed circRNA
H_2_O_2_: Hydrogen peroxide
MDA: Malondialdehyde
CAT: Catalase
T-SOD: Total superoxide dismutase
