# Supplementary material for: Multi-omics strategies uncover the molecular mechanisms of nitrogen, phosphorus and potassium deficiency responses in Brassica napus
Source: Cell Mol Biol Lett. 2023 Aug 5;28:63. doi: 10.1186/s11658-023-00479-0 (PMC10404376; doi:10.1186/s11658-023-00479-0)

**Multi-omics strategies uncover the molecular mechanisms of nitrogen, phosphorus and potassium deficiency responses in *Brassica napus***

**Ying Fu^1^, Annaliese S. Mason^3^, Maolin Song^1, 2^, Xiyuan Ni^1^, Lei Liu^1^, Jianghua Shi^1^, Tanliu Wang^1^, Meili Xiao^4^, Yaofeng Zhang^1^, Donghui Fu^4^ and Huasheng Yu^1^**

^1^ Institute of Crop and Nuclear Technology Utilization, Zhejiang Academy of Agricultural Sciences, Hangzhou, China

^2^ College of Advanced Agricultural Sciences, Zhejiang A&F University, Hangzhou, China

^3^ Plant Breeding Department, University of Bonn, Katzenburgweg 5, 53115 Bonn, Germany

^4^ Key Laboratory of Crop Physiology, Ecology and Genetic Breeding, Ministry of Education, Agronomy College, Jiangxi Agricultural University, Nanchang, 330045, China. fudhui@163.com.

* Corresponding author

Phone: +86 (0) 571 86404096; Fax: +86 (0) 571 86404096; E-mail: [yuhuasheng-0@163.com](mailto:yuhuasheng-0@163.com)

Phone: +86 (0) 791 83813185; Fax: +86 (0) 791 83813185; E-mail: [fudhui@163.com](mailto:fudhui@163.com)

Figure S1 The common miRNA-target gene pairs of rapeseed seedings responsive to different macronutrient deprivations. (a) common miRNA-target pairs involved in 'oxidation-reduction process’ responsive to both N and P deprivations in rapeseed roots. (b) common miRNA-target pairs involved in 'transmembrane transport’ responsive to both N and P deprivations in rapeseed roots. (c) common miRNA-target pairs involved in 'response to oxidative stress’ in rapeseed roots.


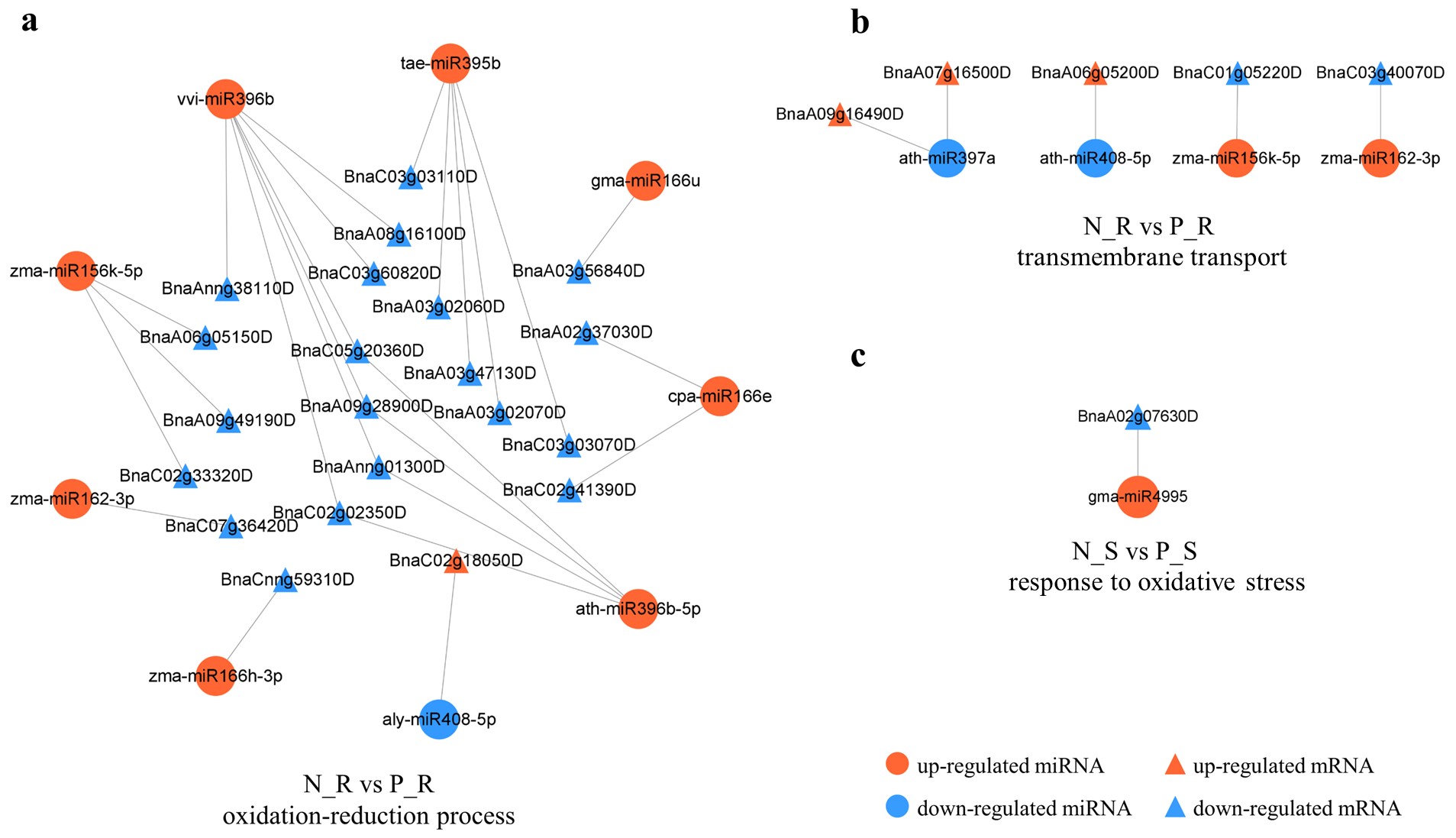


Figure S2 The validation of differentially expressed genes from RNA-seq by qRT-PCR. The [histogram](javascript:;) represented the relative expression from RNA-seq, the [curve graph](javascript:;) represented the relative expression from qRT-PCR. The [correlation coefficient](javascript:;)s between two methods were marked at the top of each chart.


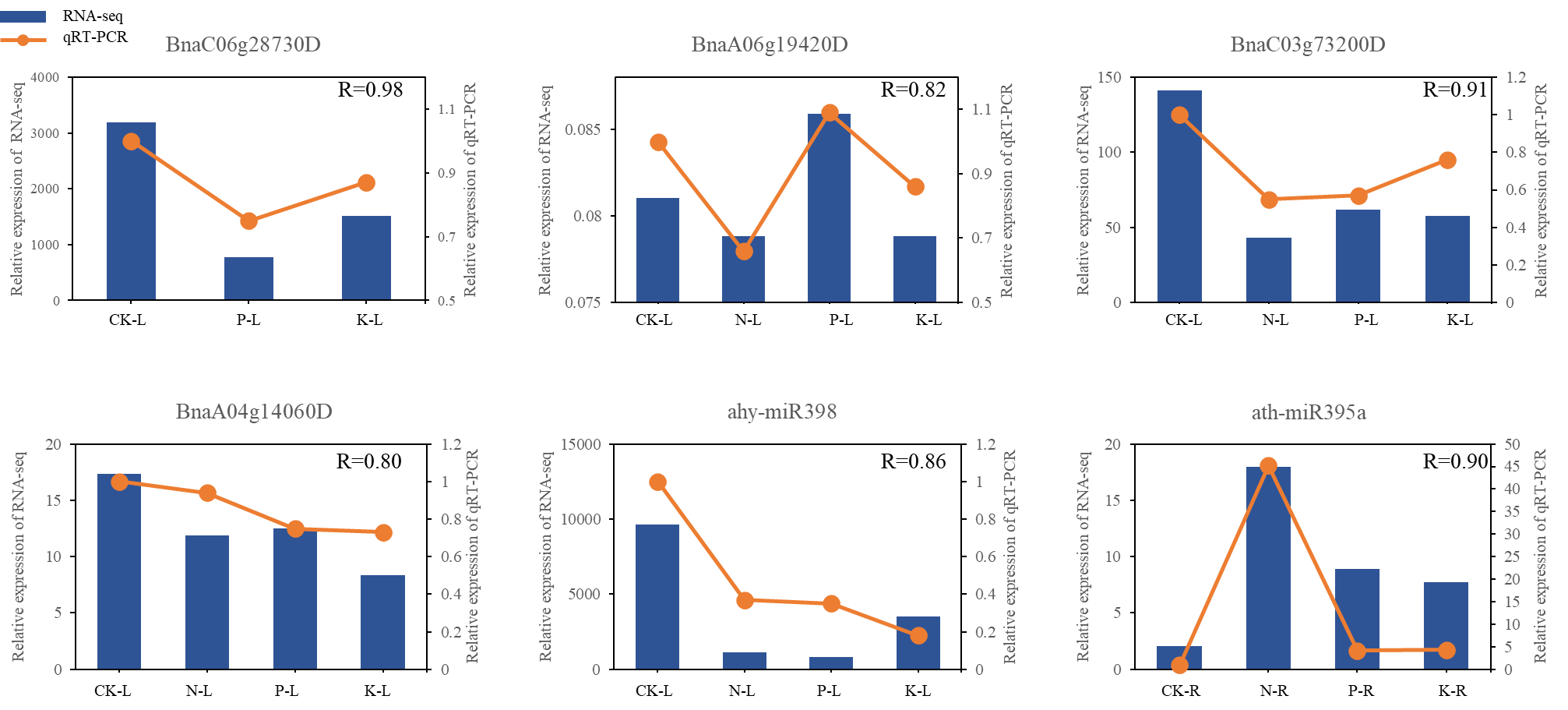


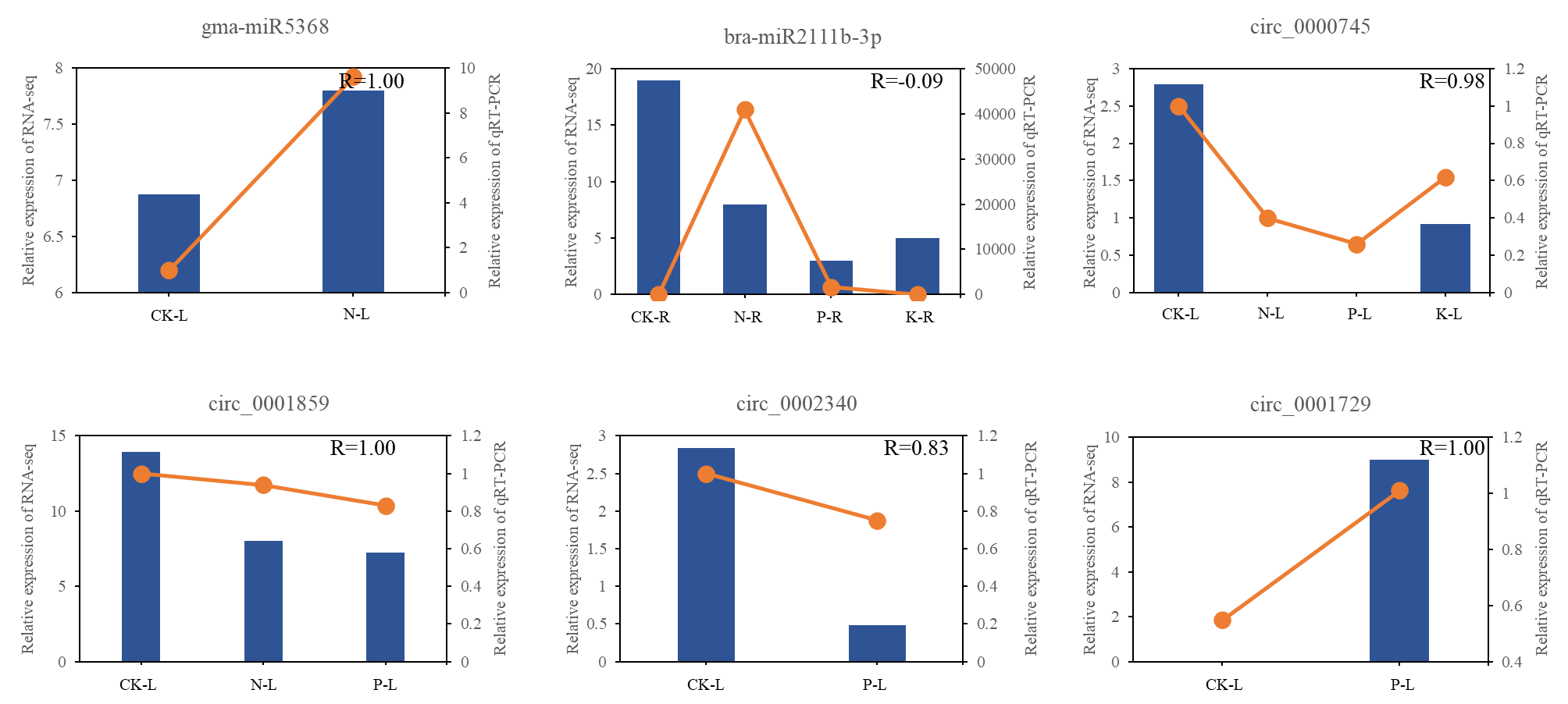

Supplement: Supplementary file 1 — Additional file 1. Supplementary Figures. [file 11658_2023_479_MOESM1_ESM.docx]
